# Neonatal intestinal colonization of *Streptococcus agalactiae* and the multiple modes of protection limiting translocation

**DOI:** 10.1080/19490976.2024.2379862

**Published:** 2024-07-23

**Authors:** Kara G. Greenfield, Olivia S. Harlow, Lila T. Witt, Evelyn M. Dziekan, Christian R. Tamar, Josephine Meier, Jane E. Brumbaugh, Emily R. Levy, Kathryn A. Knoop

**Affiliations:** a Department of Immunology, Mayo Clinic, Rochester, MN, USA; b Department of Pediatric and Adolescent Medicine, Mayo Clinic, Rochester, MN, USA

**Keywords:** Group B *Streptococcus*, Neonatal Sepsis, Intestine, Gut Microflora

## Abstract

*Streptococcus agalactiae*, also known as Group B *Streptococcus* (GBS), is a predominant pathogen of neonatal sepsis, commonly associated with early-onset neonatal sepsis. GBS has also been associated with cases of late-onset sepsis potentially originating from the intestine. Previous findings have shown GBS can colonize the infant intestinal tract as part of the neonatal microbiota. To better understand GBS colonization dynamics in the neonatal intestine, we collected stool and milk samples from prematurely born neonates for identification of potential pathogens in the neonatal intestinal microbiota. GBS was present in approximately 10% of the cohort, and this colonization was not associated with maternal GBS status, delivery route, or gestational weight. Interestingly, we observed the relative abundance of GBS in the infant stool negatively correlated with maternal IgA concentration in matched maternal milk samples. Using a preclinical murine model of GBS infection, we report that both vertical transmission and direct oral introduction resulted in intestinal colonization of GBS; however, translocation beyond the intestine was limited. Finally, vaccination of dams prior to breeding induced strong immunoglobulin responses, including IgA responses, which were associated with reduced mortality and GBS intestinal colonization. Taken together, we show that maternal IgA may contribute to infant immunity by limiting the colonization of GBS in the intestine.

## Introduction

Neonatal sepsis, often the result of bloodstream infections during the first 28 d postnatal, remains an important public health concern worldwide, as neonates have the highest incidence of sepsis compared to other age groups.^1^ Neonates born prematurely (<37 weeks gestational age) or at a very low birth weight (VLBW, <1500 g) are particularly susceptible to neonatal sepsis, with an incidence of approximately 8% and a mortality rate of 19.2%.^2–5^ Neonatal sepsis is clinically subdivided into early-onset (EOS) and late-onset neonatal sepsis (LOS), classified by the age of onset of infection. EOS is defined by a positive bacterial blood culture within the 72 hours (hrs) following delivery with some definitions including through 7 d following delivery, depending on gestational age at birth. *Streptococcus agalactiae*, also known as Group B *Streptococcus* (GBS), remains a major cause of neonatal pneumonia, meningitis, and sepsis. GBS rectovaginally colonizes 10–25% of women as part of the normal microflora, can be transmitted *in utero* or during the delivery process, and is implicated in 45% of EOS cases in term infants, and 25% of EOS cases in very low birth weight infants.^6,7^ GBS can utilize different mechanisms to gain access to the neonatal bloodstream, as it has been found to directly infect cells of the placenta,^8^ and can also invade epithelial cells of the lung following the inhalation of GBS-infected amniotic fluid.^9^ Rates of EOS have decreased after the implementation of screening for maternal GBS colonization and administration of intrapartum prophylactic antibiotics to culture-positive GBS carriers.^10–12^ Though more commonly associated with EOS, GBS is also associated with LOS.^13–17^ Multiple routes of neonatal infection by GBS can contribute to LOS, including intestinal colonization, which can occur throughout early life as neonates who are initially negative for GBS upon hospital discharge can acquire GBS colonization later.^18,19^

The malleable, rapidly changing neonatal gut microbiome can serve as a reservoir for potential pathogens. Indeed, the causative pathogen of sepsis in neonates, including GBS, can often be found in stool 24–72 hrs preceding bloodstream infection,^20,21^ highlighting the importance of healthy microbiota and dietary factors which limit pathogen presence in the intestinal tract in early life. Maternal IgA is the primary source of IgA for neonates and can shape the neonatal microbiota and provide protection from enteric pathogens as the microbiota expands in diversity.^22^ Following delivery, the relatively aseptic environment of the neonatal intestine undergoes vast immunological changes and is particularly susceptible to modulation via maternal factors; delivery route, gestational age, early life diet, and antibiotic use can each uniquely affect the diversity of the neonatal microbiota. This strong tie to clinical variables and exposures in the intensive care unit (NICU) results in a highly individualized microbial progression in preterm neonates.^23^ Consequently, prematurely born infants may have a particularly permissive environment for the colonization of potential pathogens.

Increased *Streptococcus* species have been observed in perturbed neonatal microbiotas,^23–26^ but these sequencing reports lack the resolution to identify GBS from the larger *Streptococcus* genus. In the present study, we apply long-read sequencing using the Oxford Nanopore (ONT) platform to track GBS in neonatal stool.^27^ We show that while GBS can colonize the infant intestinal tract, the relative abundance was negatively correlated with total IgA present in matched maternal milk, suggesting maternal IgA may limit GBS colonization in the neonatal intestine. In a murine model of vertical transmission of GBS, pups surviving exposure to maternal GBS retained GBS within the intestine, though GBS had limited spontaneous translocation. Maternal IgA cross-reacted to GBS, limited intestinal colonization, and vaccination of dams reduced intestinal colonization of pups while increasing cross-reactive antibodies. Ultimately, GBS translocation was limited in our model, and our data suggest that while GBS results in a potent and robust EOS infection and intestinal colonization, multiple mechanisms including maternal immunoglobulin protect offspring from LOS.

## Results

### GBS colonization in the neonatal intestine

**Figure 1.**
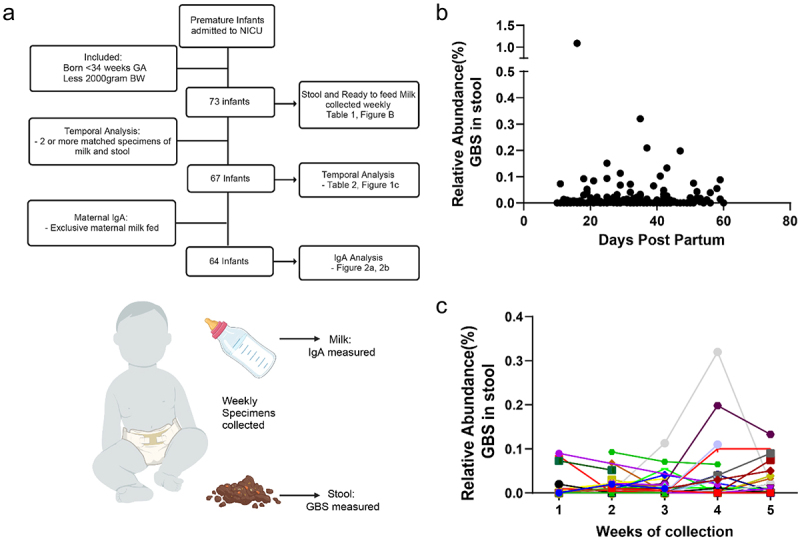
GBS is present in the stool of prematurely born infants. (a) Flow chart of study design, analysis, and specimen collection. (b) Relative abundance of GBS in stool by number of days post birth. (c) Relative abundance of GBS in stool over time of sampling in NICU cohort. *N* = 73 participants in B, *n* = 67 participants with at least 2 samples per individual C.

To determine the extent of GBS colonization in premature infants, we recruited a prospective cohort of preterm infants (<36 weeks gestational age), collecting weekly samples of milk and stool, while admitted to the neonatal intensive care unit (NICU) (Figure 1(a)). Recruitment occurred after 5 d post-partum, and collection of specimens began at 10 d post-partum or later, for 5 weeks or until discharge from the NICU. Using metagenomic sequencing of the stool from premature infants, we observed the presence of GBS in numerous individuals. GBS was found in neonates regardless of delivery route, maternal GBS status, intrapartum antibiotic administration, birth weight, gestational age, antibiotic use, and diet (Table 1). We found GBS presence in individual stool samples throughout the first 60 d post-partum with no correlation to age postpartum (Figure 1(b)). We next analyzed temporal changes in GBS abundance by assessment of the samples from infants with two or more specimens (Figure 1(c)). We found four distinct patterns: GBS undetected, initial GBS presence but later undetected, GBS presence throughout all samples, and no initial GBS followed by acquisition of GBS in latter weeks (Figure 1(c), Supplemental Table 1). Antibiotic use is common following delivery and during NICU admittance. The most common regimen of antibiotics within our cohort was empiric administration of ampicillin and gentamicin within the first 48 hrs following birth to those at risk for early-onset sepsis. However, neither this antibiotic regimen nor antibiotics administered beyond 5 d postpartum, correlated with GBS presence in the stool or temporal pattern of GBS abundance (Table S1). Thus, these data reveal that GBS colonization in premature infants in the NICU occurs frequently and can also be acquired in weeks following delivery, confirming previous observations.^17,18^

**Table 1.** Participant demographics and GBS colonization.

	Total Participants (73)	Number with GBS in at least one Stool
Delivery Route:		
Vaginal (%)	20 (26.4%)	13 (65.0%)
C-section	53 (72.6%)	39 (73.6%)
Infant Sex:		
Male (%)	34 (46.6%)	20 (58.8%)
Female (%)	39 (53.4%)	32 (82.0%)
Maternal GBS Status at time of delivery		
Positive (%)	12 (16.4%)	8 (66.6%)
Negative (%)	24 (32.9%)	17 (70.8%)
Unknown (%)	37 (50.7%)	27 (72.9%)
Maternal GBS Abx		
Treated (%)	33 (45.2%)	21 (63.6%)
Untreated (%)	40 (54.8%)	31 (77.5%)
Gestational Age (weeks)		
<32 weeks	57 (78.1%)	43 (75.7%)
>32 weeks	16 (21.9%)	9 (56.2%)
Birth Weight (grams)		
VLBW (<1500)	42 (57.5%)	27 (64.3%)
LBW (>1500, <2500)	31 (42.5%)	25 (80.6%)
Diet Throughout Study		
Maternal Milk	64	47 (73.4%)
Donor Milk	3	0 (0.0%)
Formula	4	2 (50.0%)
Infant Antibiotics		
None	15 (20.5%)	8 (53.3%)
ABX <5 d PPD	47 (64.4%)	36 (72.9%)
ABX >5 d PPD	11 (15.1%)	8 (72.7%)

**Figure 2.**
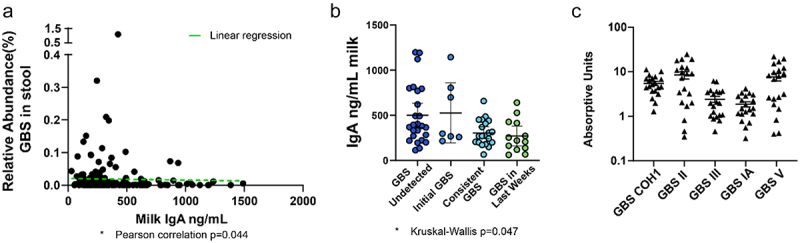
GBS colonization is associated with low dietary IgA. (a) Relative abundance of GBS in stool by amount of total IgA in diet. Pearson’s correlation *p = 0.0446 in A, linear regression (green dotted line). (b) Concentration of total IgA in milk categorized by GBS colonization patterns. “GBS undetected” = GBS undetected in all specimens, “initial GBS” = first specimen contained GBS while GBS was undetected in later specimens; “consistent GBS” = first and later specimens contained GBS, and “GBS in last weeks” = GBS was undetected in first specimens while later specimens contained GBS. Kruskal–Wallis test *p = 0.047, with no significant difference in multiple comparisons in B. *N* = 64 participants, exclusively fed maternal milk with at least 2 samples per individual in a and B. (c) GBS-cross-reactive IgA in breast milk against various GBS strains, shown in absorptive units at 450 nm. *n*= at least 20 per group in C.

As many infants in the cohort had undetectable or very minimal GBS presence in their stool, we next asked if maternal IgA may limit GBS colonization in infants. Exclusive maternal milk (“mom’s own milk”) was the predominant diet within the cohort (64/73 infants), and due to the low number of participants on an exclusive formula or donor human milk diet, we focused analysis on those exclusively fed maternal milk (Table 1). Interestingly, we observed that the relative abundance of GBS in the infant stool negatively correlated with total maternal IgA present in the matched milk (Figure 2(a)); this negative association was significant by a Pearson’s correlation (*p* = 0.044). We next assessed temporal GBS colonization in the context of total maternal IgA concentration in matched milk samples. We observed significantly different maternal IgA concentration in milk across the four patterns of temporal GBS colonization as calculated by a Kruskal–Wallis test (Figure 2(b)). While no direct comparisons were significantly different, we did observe that low IgA concentrations correlated with consistent and late GBS colonization, while increased IgA concentrations correlated with undetected or initial GBS colonization only. This trend of increased IgA concentrations associated with a lack of GBS colonization is consistent with the hypothesis that increased IgA may contribute to restricting GBS from the early-life intestinal microbiota. To determine if maternal IgA could bind GBS, we measured the amount of cross-reactivity maternal IgA had against various strains of GBS, COH1, and four clinical isolates of GBS from infant stool associated with late-onset sepsis.^20^ IgA from human milk readily bound GBS, including virulent COH1, and to differing degrees, clinical isolates from infant stool (Figure 2(c)). Thus, these data suggest maternal IgA can bind GBS and may limit GBS colonization in the maternal milk-fed infant.

### GBS pneumonia precedes EOS disease

**Figure 3.**
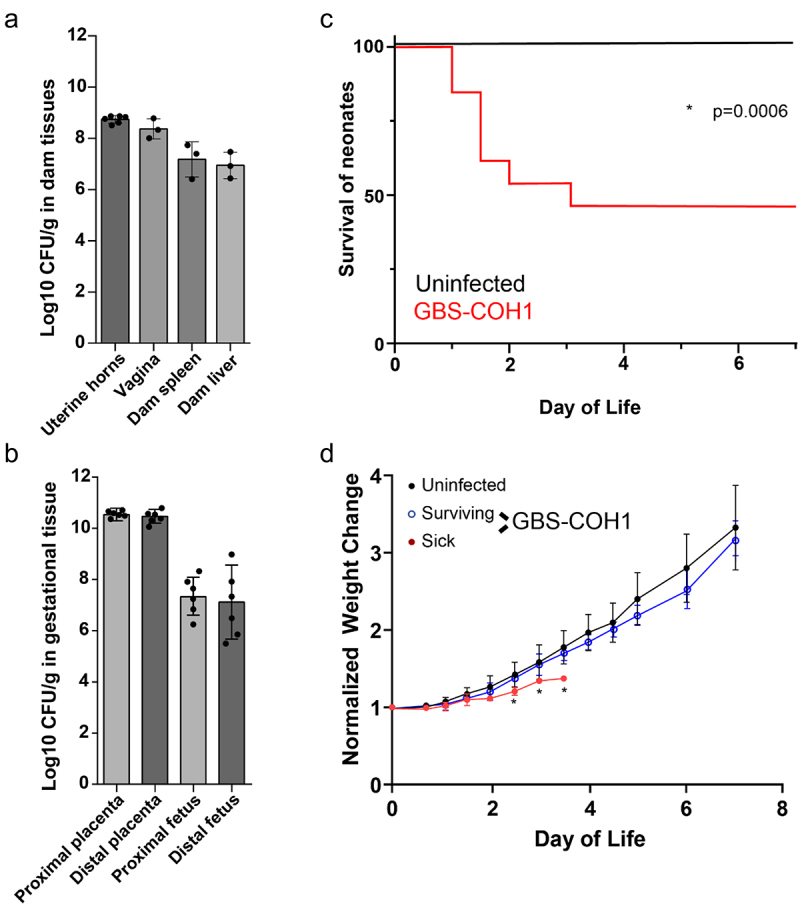
GBS is transmitted vertically during pregnancy and causes sepsis and mortality in pups. C57Bl/6 dams were vaginal colonized with10^7^ colony-forming units (CFUs) GBS COH1 on E18. (a) CFUs per gram tissue in dams on E20 following vaginal administration of GBS on E18, *n* = 3 dams. (b) CFUs per gram fetal tissue on E20, *n* = 6, 2 fetus from 3 independent dams from A. (c) Survival of pups after EOS. *n* = 3 independent litters per group with at least 5 pups at birth. *p = 0.0006 on Kaplan-Meier survival curve. (d) Weight change over time in uninfected or infected with GBS. Surviving pups were split from pups succumbing to GBS infection in C, weight gain normalized to day of life 1. Two-way ANOVA with Tukey’s multiple comparison test. *n* = 3 independent litters per group with at least 5 pups at birth from C.

We next utilized a murine model of vertical GBS transmission to explore the dynamics of GBS acquisition and colonization in offspring. Following vaginal colonization of pregnant dams on embryonic day 18 (E18), we found all placentas and fetuses, both proximal and distal in the uterine horns, contained similar pathogen burden (Figure 3(a,b)). Similar to previous reports utilizing rodent models of vertically transmitted GBS, pups rapidly succumbed to infection,^28–30^ with 50% lethality beginning 1 day following delivery through 3 days postnatal (P3) (Figure 3(c)). Comparing surviving pups to pups succumbing to infection, we observed significant weight gain in pups surviving vertical transmission of GBS, particularly at P3 and P4 (Figure 3(d)). Surviving pups appeared healthy and continued to gain weight beyond this time (Figure 3(d)).

**Figure 4.**
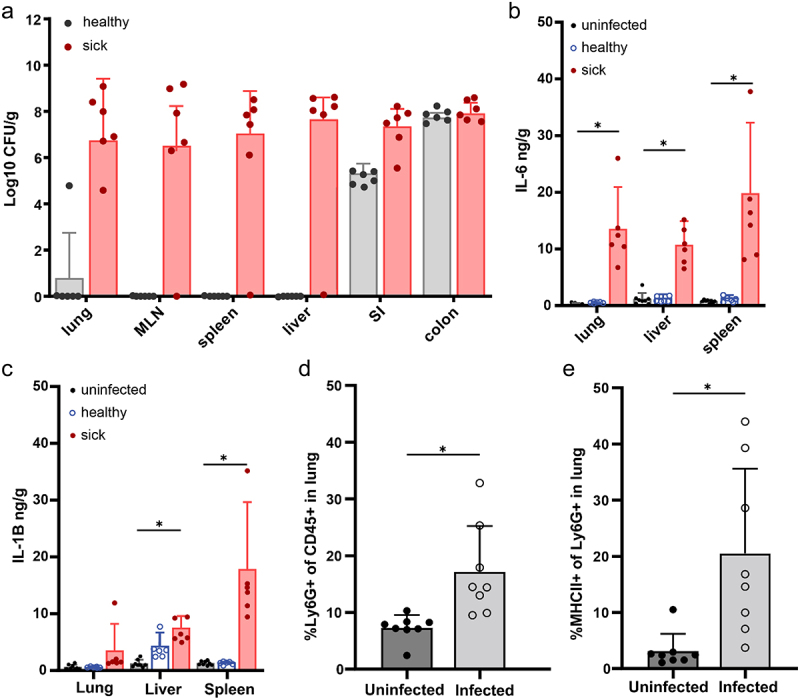
GBS EOS is associated with pneumonia. C57Bl/6 dams were vaginal colonized with 10^7^ CFUs GBS COH1 on E18. (a) CFUs per gram of organ in healthy and sick pups on P3, healthy and sick was defined by weight gain. MLN = mesenteric lymph node, SI = small intestine. (b) ELISA of tissue homogenates for IL-6 and (c) IL-1B. A–C, *n* = 6 per group, 2 sick and 2 healthy from 3 independent litters. (d) Neutrophils in the lung after EOS on P3, defined as CD45+ Ly6G+, and (e) percent of MHCII+ antigen experienced neutrophils. D-E, *n* = 8 per group, across 3 independent litters. Two-way ANOVA with Tukey’s multiple comparison test in (a-c) comparing cytokines in each organ to control (uninfected) and Mann-Whitney (d, e) with *p < 0.05.

We next assessed the systemic infection at P3 by defining those pups failing to gain weight as “sick” and those gaining weight as “healthy”. While both groups (sick and healthy pups) had colonization of GBS in the small intestine and colon on postnatal day 3 (P3), only moribund pups had GBS in extraintestinal and systemic organs (Figure 4(a)), including in the lungs, mesenteric lymph node (MLN), spleen and liver (Figure 4(a)). After this timepoint, we rarely observed CFUs of GBS in systemic organs of surviving pups, suggesting pups that survive past P3 have minimal dissemination (data not shown). The presence of GBS in systemic organs at P3 was associated with increased IL-1β and IL-6 (Figure 4(b, c)), which are associated with both clinical GBS disease and rodent models of GBS infection.^31,32^ Flow cytometric analysis of the lungs of P3 pups revealed a significant increase in the proportion of Ly6G+ neutrophils, including antigen-experienced MHCII+ neutrophils (Figure 4(d, e)) compared to uninfected pups, suggesting vertical transmission of GBS and resulting EOS disease is associated with pneumonia-like disease.

### GBS can colonize the neonatal intestine but has limited systemic translocation

**Figure 5.**
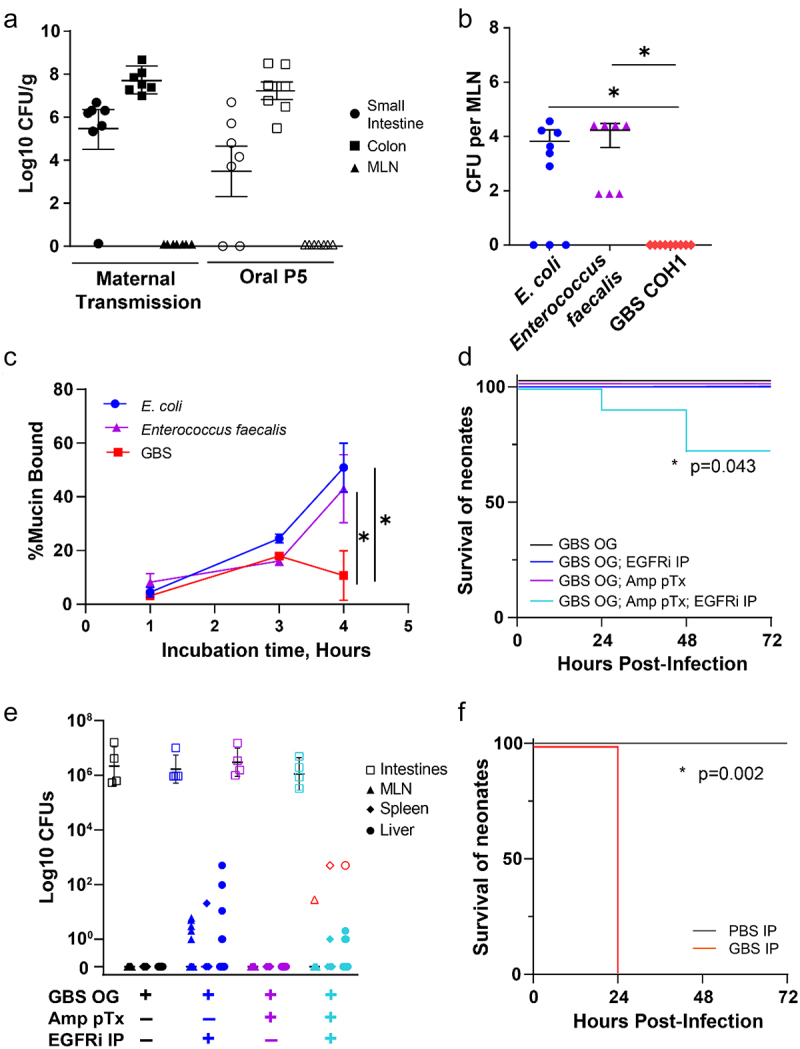
GBS colonizes the neonatal intestine and displays limited translocation. (a) CFUs in intestines and mesenteric lymph node (MLN) of 6 day old mice following: “mat Tran”= maternal transmission of GBS as in figure 4 or “oral P5” = 24 hours after gavage with 10^6^ CFU GBS. *N* = 7 per group, across 3 independent litters. (b) CFUs in the mesenteric lymph node of 6 day old mice after gavage with bacterial isolates and treatment with EGFR inhibitor (EGFRi). *N* = 6 per group. One-way ANOVA with Tukey’s multiple comparison test, *p < 0.05. (c) Relative binding of mucin to bacterial strains by contact time. two-way ANOVA (c) with Tukey’s multiple comparison test, *p < 0.05. (d) Survival of mice after oral gavage of GBS, with EGFRi, and with pretreatment of oral ampicillin. *N* = 6 per group across 3 independent litters. Kaplan-Meier curve with *p < 0.05. (e) CFUs in intestines, mesenteric lymph node, spleen, and liver 48 hours after oral gavage of GBS, with EGFRi, and with pretreatment of oral ampicillin. Red symbols indicated GBS+ translocation as identified by selective agar. *N* = 4 per group. (f) Survival of mice after intraperitoneal injection of GBS or vehicle. *n* = 5 per group. Kaplan-Meier curve with *p < 0.05.

As we observed continued GBS colonization of pups surviving maternal transmission in the intestine (Figure 5(a)), we orally inoculated pups on P5 with 10^6^ CFUs GBS, to directly expose pups to GBS postnatally independent of vertical transmission. Following oral inoculation of P5 pups, GBS readily colonized both the small intestine and colon (Figure 5(a)). In pups exposed to GBS *in utero* and postnatally, colonization of GBS in the small intestine and colon was not associated with spontaneous translocation to the MLN outside of systemic infection (Figures 4(a), 5(a)). We have previously shown that spontaneous translocation from the intestine in neonates is limited due to intestinal barrier protection provided by maternal factors in breastmilk, including epidermal growth factor (EGF).^33^ Our group has previously reported that the inhibition of epidermal growth factor receptor (EGFR) in neonates results in rapid dissemination of *E. coli*, and late-onset sepsis-like systemic infection. In this model, pathogens gain systemic access by utilizing goblet cells that form goblet-cell associated antigen passages (GAPs), as the primary route of entry. GAP formation by goblet cells in neonates is minimal, due to inhibition of this process by activation of the EGFR expressed by goblet cells by maternal EGF.^34^

Other sepsis-causing bacterial species, such as *Lactobacillus* species and *Enterococcus faecalis*, have been observed to translocate via GAPs.^35^ Thus, we wanted to test whether GBS was able to translocate through GAPs to establish a systemic infection by comparing the ability of GBS to translocate to other pathogens known to translocate via GAPs. Oral administration of *E. coli* and *Enterococcus faecalis* resulted in rapid bacterial translocation to the MLN; however, GBS was not able to translocate to the MLN following EGFR inhibition (Figure 5(b)). Goblet cells contribute to barrier protection at the intestinal epithelium by producing mucin which maintains a mucus barrier.^36^ While some pathogens can bind and cleave mucus and are able to penetrate this mucus barrier, most pathogens are restricted to the lumenal space of the intestine as a result of mucus coating the epithelium. Thus, we examined the ability of GBS to bind mucin compared to other pathogens by culturing bacterial isolates with purified mucin and assessing bacteria coated with mucin. We found that both *E. coli* and *Enterococcus faecalis* were rapidly bound by mucin; however, GBS showed a delay in and significantly less mucin binding over time (Figure 5(c)). These data suggest that GBS may have reduced capacity to invade the mucus layer overlaying the epithelium and is therefore unable to translocate systemically via goblet cells during EGFR-inhibition-induced GAP formation.

In addition to maternal factors, such as EGF, the microbiota provides an important source of protection against enteric pathogens, as previous observations found antibiotic-induced dysbiosis in neonates resulted in rapid dissemination of *Klebsiella* in a model of LOS.^37^ However, we found that treatment of mice with ampicillin prior to oral administration failed to enhance GBS translocation to MLN (Figure 5(d)). Treatment with both EGFR inhibitor and antibiotics prior to GBS inoculation resulted in the translocation of multiple bacterial species to the MLN and liver, including GBS (Figure 5(e)). However, translocation of GBS was limited and inconsistent, and did not correlate with increased mortality in pups, perhaps due to low pathogen burden via this route, as intraperitoneal (IP) GBS infection, also at P5, causes rapid mortality (Figure 5(f)). Taken together, these data suggest GBS can colonize the neonatal intestine, but can only translocate systemically following multiple disruptions to the intestinal barrier.

### Maternal vaccination decreases GBS colonization in pups and improves EOS survival

**Figure 6.**
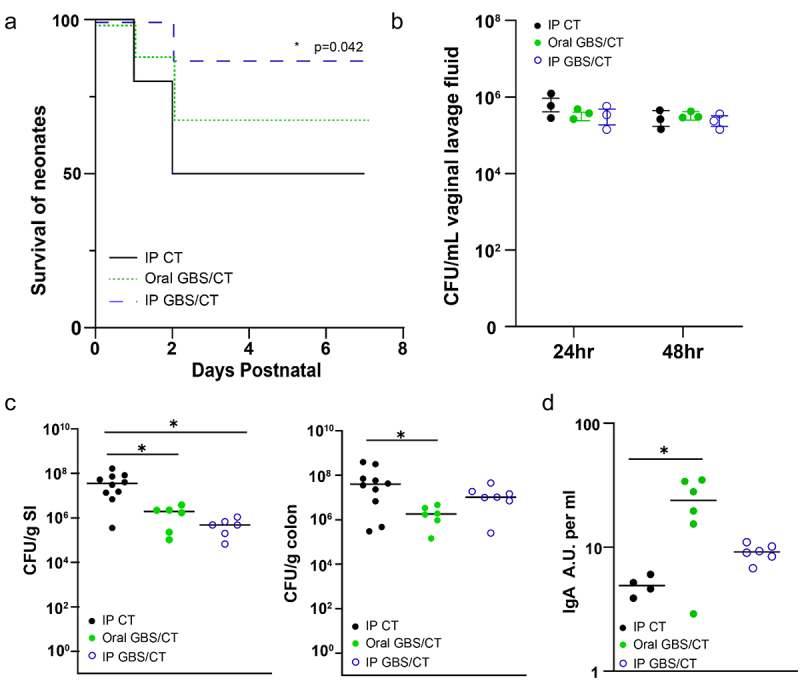
Maternal vaccination decreases GBS colonization in pups and improves EOS survival. Dams were immunized with ethanol-killed GBS and cholera toxin (CT) or CT alone, and then bred and colonized with GBS on E18. (a) Survival of pups during EOS after maternal vaccination prior to pregnancy. Vaccination with ethanol-killed GBS COH1 and cholera toxin delivered orally or intraperitoneally, or cholera toxin alone IP. *N* = 3 dams per group, with subsequent litters. Kaplan-Meier survival curves with * *p* < 0.05. (b) Colonization by GBS COH1 in the vaginal tract of pregnant mice after inoculation at ED18 in mice orally treated with cholera toxin or ethanol-killed GBS with cholera toxin. *N* = 3 dams per group. (c) CFUs in small intestinal (SI) and colonic contents of pups at PN7 after maternal vaccination prior to pregnancy. (d) Relative amount of GBS-binding IgA in the stomach contents of pups after maternal vaccination, measured in absorptive units. One-way ANOVA (C,D) and with *p < 0.05 comparing to control (IP CT).

To understand how maternal immunoglobulin may be optimized to limit GBS disease in offspring, we immunized dams with ethanol-killed GBS and cholera toxin either intraperitoneally (IP) or orally. Maternal vaccination significantly improved the survival of offspring during GBS EOS infection (Figure 6(a)), which correlated with a modest, but not significant reduction in CFU burden in systemic organs at P3 (Supplemental Figure 1). However, neither vaccination strategy impacted GBS colonization of the vaginal tract (Figure 6(b)), suggesting the protection conferred to offspring was not due to decreased pathogen transmission or exposure. While maternal immunization may provide several modes of protection, we assessed the cross-reactivity of maternal IgA to GBS from the stomach contents of pups and observed significant increase in cross-reactivity of maternal IgA from orally immunized dams (Figure 6(c)). Assessment of offspring on postnatal day 7 (P7) revealed that maternal vaccination was sufficient to limit intestinal GBS colonization in the small intestine following IP immunization (Figure 6(d)) and in both the small intestine and colon following oral immunization (Figure 6(d)). Thus, while GBS can readily colonize both the human and murine intestinal tract, maternal IgA may limit its colonization.

## Discussion

Here, we show that GBS is able to colonize the neonatal intestine following vertical transmission and direct postnatal oral exposure. Vaginal colonization of pregnant dams resulted in rapid *in utero* exposure of the fetuses to GBS; however, while all placentas and fetuses were equally exposed to GBS, 50% of pups only had intestinal colonization at P3, whereas remaining littermates succumbed to early-onset systemic infection. Thus, it is unclear if these pups can clear the systemic GBS infection and only maintain intestinal colonization, or if these pups were never subject to systemic disease. All pups succumbing to EOS following delivery had GBS in the lungs, and GBS was undetected in the systemic organs of surviving pups. Since all fetuses are equally exposed to GBS *in utero*, yet only 50% of pups develop lung infections, it is unclear if *in utero* transmission of GBS or inhalation of GBS during vaginal delivery results in a fetal infection that continues following delivery. GBS presence in the amniotic fluid may be an additional route of infection as fetuses often uptake amniotic fluid through fetal swallowing.^38^ In the lungs of pups with EOS, we observed a significant increase in neutrophil infiltration and inflammatory cytokine production compared to uninfected pups, suggesting GBS-induced EOS is associated with acute lung infection that disseminates systemically. Primary lung infection preceding systemic dissemination validates *in vitro* findings that GBS can damage lung tissue.^9^ As pregnant dams maintain vaginal colonization throughout the experiment, pups may receive a second inoculum of GBS during the delivery process, in addition to *in utero* exposure. Exposure of the respiratory tract to GBS during delivery would be consistent with a lung infection that results in EOS following delivery. Following vertical transmission of GBS, we predominantly examined the lungs for inflammation; however, we did observe GBS in the spleen, liver, and mesenteric lymph node, and increased IL-6 and IL1β in the spleen and liver, suggesting systemic and bloodstream inflammation may also be contributing to disease. We did not assess the intestine for pathology or inflammation following EOS, though it would be difficult to ascertain if potential inflammation would be the result of local or systemic GBS.

The gastrointestinal tract represents a second route by which neonates are vulnerable to pathogen entry, as the neonatal GI tract was readily colonized by GBS following either perinatal or postnatal exposure. The causative pathogens of neonatal sepsis are commonly gut-originating, including *Escherichia coli*, *Klebsiella* species, *Enterococcus faecalis, Staphylococcus aureus* and, *Streptococcus* species, particularly in preterm infants.^20,21^ In our preclinical model, direct oral GBS administration on P5 resulted in colonization of the neonatal GI tract with a similar CFU burden to intestinal GBS colonization following maternal vertical transmission. In our clinical cohort, we observed GBS presence in approximately 60% of neonates, though this was varied between neonates with individual stools with GBS detected and neonates with consistent GBS colonization. This suggests exposure to GBS, either from maternal transmission or the postnatal nosocomial environment, can result in intestinal colonization in the offspring. One potential mechanism limiting GBS colonization uncovered in our studies is through maternal IgA. In our clinical cohort, GBS abundance was negatively correlated to total IgA within maternal milk, suggesting maternal IgA limits GBS in the infant microbiome. This, taken together with the cross-reactivity of IgA from human milk to various GBS isolates, suggests natural reactivity of maternal IgA to GBS. Clinical findings have previously correlated maternal milk with protection from *Streptococcus* species, as formula feeding has been associated with increased presence of *Streptococcus*, though sequencing was performed at the genus level and could not assess GBS specifically.^25,26,39,40^ Additionally, previous reports have suggested maternal milk may serve as a source of GBS; however, we did not perform sequencing on the matched milk samples in our clinical cohort.^18^

Mammary IgA responses can be influenced and informed by the maternal intestinal microbiota^41^; though others have not observed a significant increase in cross-reactivity of maternal IgA to GBS from individuals screening positive for GBS to those who are GBS negative, these screens are based on superficial rectum swabs.^42^ Deeper sequencing of the maternal intestinal microbiome would have the power to ask if mothers colonized with GBS produce higher affinity IgA for GBS due to natural exposure and the gut-mammary axis.^43^ Furthermore, maternal vaccination may improve this maternal immunoglobulin response to improved neonatal protection,^44^ as we observed in our animal model. Maternal vaccination was sufficient to reduce GBS colonization in the offspring. Interestingly, oral maternal vaccination induced greater cross-reactive IgA production compared to intraperitoneal vaccination. Future studies will be required to assess the specific function of maternal IgA to neonatal protection by improving pathogen exclusion from the microbiota.

Maternal vaccination strategies capable of reducing early-onset GBS infections in neonates have been deemed a priority by the World Health Organization.^45^ In preclinical models, immunizing dams with a GBS polysaccharide resulted in increased GBS-specific IgG in offspring, which limited GBS colonization following intranasal infection and was sufficient to reduce weight loss following systemic GBS challenge.^46^ In a model using intranasal maternal vaccination with whole-cell GBS, high titers of GBS-specific IgG antibody were generated; however, this strategy did not reduce long-term intestinal colonization or mortality in offspring.^30^ Our study suggests a potential role for enhanced maternal IgA production in reducing the ability of GBS to colonize the neonatal intestine, if IgG is not sufficient to reduce intestinal colonization of GBS in neonates. Maternal IgA has also been shown to play a protective role in binding pathogens and limiting disease in other early life infections, such as necrotizing enterocolitis (NEC). Clinical studies have reported a negative correlation between IgA bound bacteria and development of NEC, with premature infants who developed NEC exhibiting a lower level of IgA bound bacteria compared to infants who did not develop NEC.^22^ Our studies did not assess maternal IgG, and the reduction in mortality from early-onset disease following maternal vaccination could be due to increased specific IgG responses. However, we hypothesize that improved maternal IgA specificity contributes to reduced intestinal colonization, particularly following oral vaccination as the oral route is strongly connected to the development of IgA responses.^47^ Taken together, our preclinical and clinical data implicate the role of maternal IgA in providing critical protection from early life infection. Improving the specificity of maternal IgA to limit GBS colonization may be a benefit of maternal vaccination against GBS.

Beyond maternal components of protection, the neonatal intestine has multiple mechanisms that limit systemic pathogen dissemination.^48^ The mucus barrier coating the intestinal epithelium dynamically develops in the fetal intestine and postnatally, and contributes to protection from pathogens including in neonates.^49^ It has been proposed that the intestinal barrier of rodents at term is underdeveloped compared to humans at term, and a mouse at P5-P7 resembles the intestine of prematurely born infants, highlighting the need for models replicating the developmental stage most at risk for neonatal sepsis.^50^ Goblet cells form GAPs as part of the mucus secretion process,^50^ and have been shown to facilitate the translocation of certain bacterial species.^35^ Enteric bacteria have varying affinities and interactions with intestinal mucus, with some species bound by mucins and others able to cleave mucins, which may contribute to invasion of the mucus layer and systemic bacterial translocation. Indeed, increased disease resulting from a hypervirulent isolate of GBS was associated with improved ability to bind/adhere to intestinal epithelial cells.^51^ Additionally, the impact of circulating hormones following birth may impact the virulence of GBS and physiology of the intestinal barrier.^52^

We found that both *E. coli*, as previously reported, and *Enterococcus* were able to translocate to the MLN following EGFR-inhibition-induced GAP formation, and exhibited greater ability to bind mucin compared to GBS. Our mucin-binding assay utilized porcine mucin and future work should seek to understand how pathogens interact with the neonatal mucus barrier, particularly in the mucus barrier of prematurely born infants. In contrast to the severity of GBS disease in early-onset disease following vertical transmission, we observed relatively mild intestinal-associated GBS disease. GBS was only able to translocate to MLN following both EGFR-inhibition-induced GAP formation and ampicillin pretreatment, thus only resulting in minimal late-onset disease. The inability of GBS to cause systemic disease can be attributed to multiple factors restricting translocation of GBS, including IgA, mucus, and microbial competition.

The microbiota also contributes to limiting systemic bacterial translocation by promoting intestinal barrier integrity. Colonization of the intestinal tract with GBS has also been shown to lead to meningitis in neonates due to the immaturity of the microbiota, resulting in a more permissive intestinal barrier.^53^ In the absence of an intact, healthy microbiota, GBS may be able to access systemic circulation via multiple intestinal pathways, including GAPs and tight junctions.^53^ The ability of GBS to translocate to MLN following antibiotic treatment highlights the influence of the microbial landscape on the ability of GBS to maintain a niche and disseminate from the intestine and may promote its continued presence and potential pathogenicity.^54^ *Lactobacillus* strains have antagonistic effects on GBS colonization,^55^ and maternal probiotic interventions utilizing *Lactobacillus* reduced GBS colonization postpartum.^56^ Further investigation is necessary to explore the potential competition and other factors microbes face when accessing GAPs for lumenal translocation. Prematurely born infants within the NICU often receive multiple courses of antibiotics, which has drastic effects on the developing microbiota.^57,58^ Additionally, intrapartum antibiotics for the prevention of vertical GBS transmission can disrupt the neonatal microbiota.^59^ Throughout early life, the microbiota rapidly expands in diversity and complexity in what is usually a controlled, systematic progression; however, preterm neonates experience delayed colonization by bacteria such as *Bifidobacterium* and *Bacteroides* species, which are integral aiding digestion of complex carbohydrates, as well as limiting colonization by potentially pathogenic bacteria.^57,60,61^ This delayed colonization impacts the progression of the microbiota, resulting in a distinct microbial signature that can be seen up to 4 years of age.^62^ Additionally, while efforts are made to provide premature infants with breast milk as their primary diet, neonates may not have access to breast milk or may be parenterally fed. Thus, premature infants in the NICU may have multiple sources of vulnerability to intestinal disease: antibiotic-induced dysbiosis, with a lack of dietary EGF, and continued exposure to opportunistic pathogens.

In conclusion, we find multiple routes of neonatal GBS exposure results in a long-term intestinal colonization. GBS has the potential to translocate from the intestine, given multiple disruptions to the intestinal environment. Thus, multiple components protect the vulnerable neonate from GBS-induced LOS, including components found in maternal milk, such as IgA and EGF. Preclinical models suggest maternal vaccination against GBS could protect against EOS, suggesting improved maternal IgA responses may improve disease outcomes in offspring. Our findings contribute to understanding the distinct ways that GBS may induce disease in early life and suggest promotion of human-milk-based diets and maternal vaccination may reduce GBS intestinal colonization in neonates.

### Limitations of this study

Due to the design of the clinical cohort, we were unable to sample meconium or stool from infants immediately following parturition. Therefore, we are unable to assess GBS colonization in the first week postpartum. Some of the infants with GBS present in their stool were born to mothers with negative GBS swabs and had multiple collection points that were GBS negative prior to GBS presence in the stool. Therefore, we interpret this data to be supportive that GBS can be acquired weeks following delivery, potentially environmentally acquired. However, more work is needed to assess the role of GBS acquisition at birth compared to post-delivery acquisition of GBS. Additionally, while the identification of GBS relied on metagenomic sequencing of the infant stool, we have yet to fully characterize this dataset to look at the abundance of other key sepsis pathogens. This report is focused on *Streptococcus agalactiae*, and the larger metagenomic dataset will be assessed in the future work. As we found *E. coli* and *Enterococcus faecalis* also readily translocated through goblet cells in our murine model, it will be of interest to see how these species interact with GBS in the human intestinal microbiota. Finally, within our cohort, no cases of LOS occurred, therefore our cohort is unable to assess the contribution of maternal IgA from LOS resulting from GBS. While we did observe a protective role of maternal IgA from GBS colonization, future work connecting this role to neonatal LOS outcomes is required.

## Materials and methods

*Human Research*: 73 premature/low birth weight neonatal participants less than 34 weeks gestational age and/or less than 2000 g birth weight were recruited following informed consent. Weekly samples of diet and stool were collected for up to 5 weeks while at Mayo Clinic Hospital Neonatal Intensive Care Units. Participants discharged prior to this time were included in the study. Samples were collected by nursing staff: stool from the diaper and “ready to feed” milk from the bottle aliquot just prior to feeding. Clinical metadata was collected including route of delivery, sex, gestational age, birth weight, and antibiotic use. Maternal GBS status at the time of delivery was defined as “positive” and “negative”, as determined by rectal swab, or “unknown” if no test was administered. Empiric maternal intrapartum antibiotic administration as a result of GBS status was categorized in “Maternal GBS Abx”. Infant antibiotic exposure was categorized as “No abx” = no antibiotics administration, “<5 PPD” = antibiotics were administered in the first 5 d postpartum, “>5 PPD” = antibiotics were administered beyond 5 d postpartum. The predominant regimen of antibiotics was <5 PPD, and typically ampicillin/gentamicin in the first 48 hrs following delivery as indicated. Diet throughout the study was noted by nursing staff. All participants in this study were exclusively fed either maternal milk, donor human milk, or infant formula. IRB approval was provided by Mayo Clinic, 20–012814.

*GBS identification from stool*: DNA was extracted from stool using the Qiagen Power Fecal Pro DNA Kit (Qiagen, #51804). DNA was fragmented and barcoded using the Oxford Nanopore (ONT) rapid PCR barcoding kit (Oxford Nanopore, #SQK-RPB004). Libraries were then sequenced in an Oxford Nanopore GRIDion, and downstream analysis was performed using centrifuge.^27^ The relative abundance of GBS is reported and calculated from dividing total reads by sequences aligning to the GBS genome. Temporal pattern of GBS colonization was categorized as “GBS Undetected” = GBS undetected in all specimens, “Initial GBS” = first specimen contained GBS, while GBS was undetected in later specimens; “Consistent GBS” = first and later specimens contained GBS, and “GBS in last weeks” = GBS undetected in first specimens, while later specimens contained GBS.

*Mice*: C57Bl/6J mice were purchased from The Jackson Laboratory and bred and maintained in-house. Mice were GBS-free at the time of arrival, and all mice were monitored for GBS colonization throughout the studies. Mice were used for colonization between 13 and 30 weeks of age. Female mice were bred in harem with a male mouse, and harems were separated after 14 d. All experiments were repeated on at least three independent dams and their litters. All litters remained in the cage with the dam and were nursed by the dam that delivered the litter. No litters were fostered to different dams. All pups from a litter were analyzed. Only the first litter of dams were used. Animal procedures and protocols were performed in accordance with the Institutional Animal Care and Use Committee at Mayo Clinic.

*Bacterial culture conditions*: Group B *Streptococcus* strain COH1 (ATCC #BAA-1176) is a capsular serotype III ST-17 beta-hemolytic clone obtained from neonate with a bloodstream infection. COH1 was used exclusively for murine experiments. Clinical isolates were obtained from neonates with a bloodstream infection.^20^ GBS was grown in LB broth (Invitrogen, Thermo Fisher Scientific) and on tryptic soy agar (TSA, Difco Laboratories) at 37°C in 5% CO_2_.

*ELISAs*: The following ELISA kits were used according to manufacturer specifications: from Invitrogen Human IgA (#88-50,600-88), Mouse IgA (#88-50,450-77), Mouse IL-6 (#88-7064-77), Mouse IL-1β (#88-7013-77). Absorbance values were measured using a BioTek 800TS microplate reader. Tissue homogenates from the EOS model were saved and stored at −20°C. Tissues were then thawed for IL-1β and IL-6 analysis by ELISA (Invitrogen).

*Cross-reactive IgA ELISAs*: Overnight GBS cultures were subcultured 1:50 and grown to early-log phase growth (OD_600_ = 0.3) in LB broth. Ten-milliliter GBS was centrifuged at 10,200×g for 30 min and washed with 1 mL PBS. GBS was resuspended in 4% Paraformaldehyde and incubated at room temperature for 30 min. GBS was centrifuged at 8000×g for 8 min and washed with 1 mL PBS twice. Fixed bacteria was diluted to a total volume of 10 mL for approximately 10^8^ CFU/mL. 96 well flat-bottom plate was coated using 100 μL fixed bacteria per well and incubated overnight at 4C then blocked using PBS with 10% BSA. Human milk samples were diluted, plated in triplicate, and incubated overnight at 4C. Components from a Human IgA ELISA kit (Invitrogen #88-50,600-88) were used to complete the ELISA protocol.

*Vertical transmission model of early-onset sepsis*: Overnight GBS COH1 cultures were subcultured 1:50 and were grown to mid-log phase growth (OD_600_ = 0.5) in LB broth. One-milliliter GBS was centrifuged at 8000×g for 8 min and washed with 1 mL PBS. Finally, GBS was resuspended in 500 µL PBS (~10^7^ CFU/10 µL). Pregnant mice at gestational/embryonic day 18 (ED18) were anesthetized with 3% isoflurane, then were vaginally inoculated by pipetting 10 µL bacteria 5 mm deep into the vagina. Mice were left inverted under anesthesia for 5 min. Vaginal lavages were collected E19 and E20 by dispensing 50 µL sterile PBS with a micropipette into the vagina and removing and reintroducing the PBS 10 times. This was repeated at least three times for a total of 150 µL of recovered vaginal lavage. Vaginal lavages were serially diluted, plated on CHROMagar StrepB agar (CHROMagar #SB282), and incubated overnight at 37 °C to quantify colonies of GBS. Once pups were born, they were checked every 12 hrs to assess weight the first 3 d of life. The weight gain was normalized to the initial birthweight. From PN4-PN7, pup weight was checked every 24 h. If pups lost more than 0.05 g from a previous weigh-in, they were euthanized as per IACUC protocol. Pups failing to gain weight were defined as “sick”, while pups gaining weight at each timepoint were defined as “healthy”. To assess bacterial colonization, the small intestine, colon, mesenteric lymph node, spleen, liver, and lungs were aseptically removed and homogenized in PBS. The homogenates were serially diluted and plated for CFU counts on CHROMagar StrepB. In some experiments, spleen, liver, uterine horns, proximal and distal fetuses and placentas from dams at E20 following euthanasia were homogenized in PBS, serially diluted, and plated on CHROMagar StrepB to assess colonization *in utero*. The innoculum of GBS in this vertical transmission model was titered for an LD50 by 2–3 postnatal days.

*Flow cytometry*: Lungs from PND3 mice were mechanically homogenized and digested in 400ul RPMI containing collagenase II and IV for 30 min at 37°C. Cells were then filtered, spun down, and resuspended in 200 ul FACS buffer (PBS containing 5% human serum, 0.5% BSA, 0.1% sodium azide) and allowed to be blocked for 20 min at 4°C degrees. Surface master mix was made in FACS buffer, and surface staining was performed for 30 min in the dark at 4°C degrees. Following this staining, samples were washed twice with FACS buffer, and samples were acquired on an Attune NXT flow cytometer (Invitrogen). Neutrophils were identified as CD45+Ly6G+ cells. Activated neutrophils were defined as CD45+ Ly6G+ MHCII+.

*Vaccination*: GBS COH1 was cultured as described above. After centrifugation and washing, 10^7^ CFUs GBS were killed by resuspending in 70% ethanol for 15 min. The bacteria was centrifuged, washed twice with PBS, and plated on TSA to confirm lack of viability. Cholera toxin (CT) from *Vibrio cholerae* (Millipore Sigma #C8052) was used as an adjuvant to induce a mucosal IgA immune response. Female mice were vaccinated, either via oral gavage or intraperitoneal injection, with 10^8^ CFUs killed GBS + 20ug CT between 6 and 8 weeks of age, with a second dose 3 weeks after the first dose. Mice were mated 1 week after the booster dose. An early-onset sepsis model beginning on ED18 was followed as described above. Vehicle immunized mice were immunized with CT in PBS as a control.

*Late-onset sepsis model*: Overnight GBS cultures were subcultured 1:50 and grown to early-log phase growth (OD_600_ = 0.3) in LB broth. Ten-milliliter GBS was centrifuged at 10,200×g for 30 min, then resuspended in 1–10 mL PBS. Mice at PN5 were colonized via oral gavage with 10^6^ CFU/50 µL PBS and were injected intraperitoneally with 500 μg/kg tyrphostin AG1478 (epidermal growth factor receptor inhibitor, EGFRi) (Sigma Aldrich #T4182). EGFRi was initially diluted in dimethyl sulfoxide (DMSO) (Santa Cruz Biotechnologies #sc -358,801) to a concentration of 3.3 mg/mL, then further diluted in phosphate buffered saline (PBS) (Gibco #10–010) to a concentration of 33 μg/mL. Mice were injected with 16.6 μL per gram bodyweight with EGFRi to enable bacterial translocation from the gut. Mice were monitored until PN7, when they were sacrificed, and bacterial colonization of small intestine, colon, mesenteric lymph node, and spleen was assessed as described above. The innoculum of GBS in oral administration was titered for similar colonization as the EOS model above.

*Mucin Binding Assay*: Bacterial binding to mucins was assessed in a protocol modified from Grigoryeva et al. 2021^63^. Briefly, GBS and *E. coli* were grown to log phase growth (OD_600_ = 0.3). Bacteria was centrifuged at 10,200×g for 30 min and resuspended in 1 mL PBS, then stained with 2 μL of 5 mM SYTO™ BC Green Fluorescent Nucleic Acid Stain (ThermoFisher #S34855) in the dark at 37 °C for 30 min. Bacteria was centrifuged at 10,200×g for 8 min and resuspended to 800 μL in 50 mM carb/bicarb buffer. Two hundred microliter stained bacteria was then added to 800 μL of 100 µg/mL porcine MUC native protein (MyBioSource #MBS5311023) in 50 mM carb/bicarb buffer and incubated for 1–6 h, then stained with rabbit anti-porcine MUC2-CF405M (Biorbyt #ORB372331) in the dark at 37 °C for 30 min. Controls included unstained bacteria, SytoBC-stained bacteria, MUC2-CF405M + UltraComp eBeads™ Flow Cytometry compensation beads (Invitrogen #01-2222-42), SytoBC-stained bacteria + MUC2-CF405M (no mucin). The analysis consisted of dividing double positive populations in experimental samples by double positive populations in SytoBC-stained bacteria + MUC2-CF405M (no mucin) controls for relative binding.

*Statistics*: All statistical analysis was performed using GraphPad Prism 10.0 (GraphPad Software Inc.) and included One-way ANOVA, Two-way ANOVA, Mann-Whitney, Kruskal-Wallis and Pearson's correlation as noted in the figure legends. ANOVA was followed with Tukey’s multiple comparisons test. Significance was defined as *p* < 0.05. Kaplan–Meier survival curves are reported as a percentage of surviving individuals per 12-hr increments. Data is reported as mean ± standard error of the mean or standard deviation as noted in the figure legends.

## Supplementary Material

Supplemental Material

## Data Availability

All data supporting the findings of this study are available within the article.
