# Supplementary material for: Neonatal intestinal colonization of Streptococcus agalactiae and the multiple modes of protection limiting translocation
Source: Gut Microbes. 2024 Jul 23;16(1):2379862. doi: 10.1080/19490976.2024.2379862 (PMC11268251; doi:10.1080/19490976.2024.2379862)
Supplement: Supplemental Material [file KGMI_A_2379862_SM5703.zip › Supplementary files.docx]

|  | No Abx | <5 PPD | >5 PPD | Total |
| --- | --- | --- | --- | --- |
| GBS Undetected | 7 | 13 | 5 | 25 |
| Initial GBS | 1 | 6 | 0 | 7 |
| Consistent GBS | 3 | 17 | 2 | 22 |
| GBS in Last Weeks | 2 | 8 | 3 | 13 |
| Total | 13 | 44 | 10 | 67 |

**Supplemental Table 1: Antibiotics and Temporal GBS Colonization Pattens**. Temporal pattern of GBS colonization was categorized as “GBS Undetected” = GBS undetected in all specimens, “Initial GBS” = first specimen contained GBS while GBS was undetected in later specimens; “Consistent GBS” = first and later specimens contained GBS, and “GBS in last weeks” = GBS was undetected in first specimens while later specimens contained GBS. Infant antibiotic exposure was categorized as “No abx” = no antibiotics administration, “<5 PPD” = antibiotics were administered in the first 5 days postpartum, “>5 PPD” = antibiotics were administered beyond 5 days postpartum. No significant difference was observed by Fisher’s exact test.


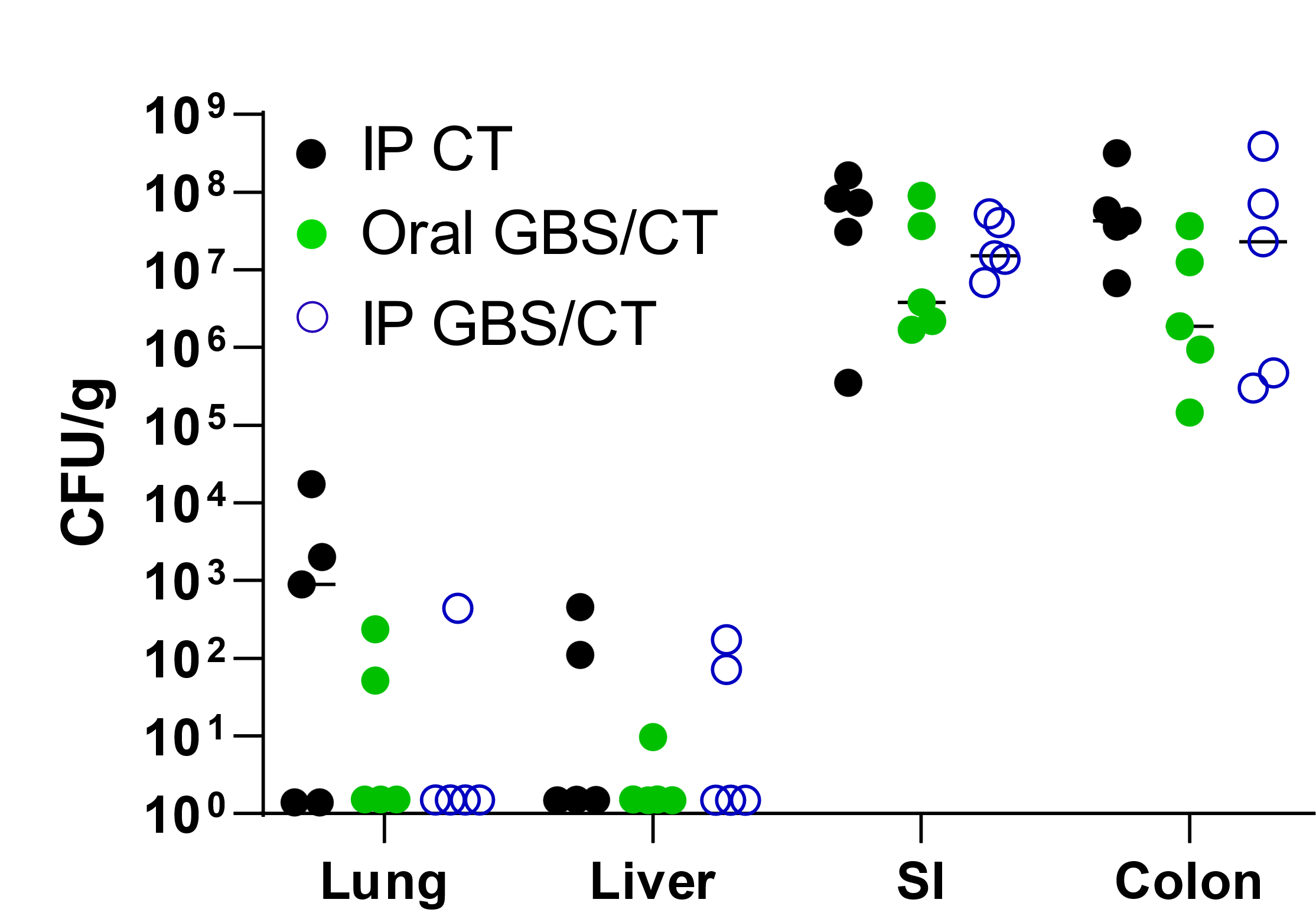


Supplemental Figure 1: Dams were immunized with ethanol-killed GBS and cholera toxin (CT) or CT alone, and then bred and colonized with GBS on E18. CFUs in lung, liver, small intestinal and colonic contents of pups at PN3 after maternal vaccination prior to pregnancy. N=5 pups per each group.
